# CRISPR-dCas9 based DNA detection scheme for diagnostics in resource-limited settings†

**DOI:** 10.1039/d1nr06557b

**Published:** 2022-01-19

**Authors:** Michel Bengtson, Mitasha Bharadwaj, Oskar Franch, Jaco van der Torre, Veronique Meerdink, Henk Schallig, Cees Dekker

**Affiliations:** a Department of Bionanoscience, Kavli Institute of Nanoscience Delft, Delft University of Technology Delft the Netherlands C.Dekker@tudelft.nl; b Amsterdam University Medical Centers, Academic Medical Centre at the University of Amsterdam, Department of Medical Microbiology and Infection Prevention, Laboratory for Experimental Parasitology, Amsterdam institute for Infection and Immunity Amsterdam the Netherlands

## Abstract

Nucleic-acid detection is crucial for basic research as well as for applications in medicine such as diagnostics. In resource-limited settings, however, most DNA-detection diagnostic schemes are inapplicable since they rely on expensive machinery, electricity, and trained personnel. Here, we present an isothermal DNA detection scheme for the diagnosis of pathogenic DNA in resource-limited settings. DNA was extracted from urine and blood samples using two different instrument-free methods, and amplified using Recombinase Polymerase Amplification with a sensitivity of <10 copies of DNA within 15 minutes. Target DNA was bound by dCas9/sgRNA that was labelled with a DNA oligomer to subsequently induce Rolling Circle Amplification. This second amplification step produced many copies of a G-quadruplex DNA structure that facilitates a colorimetric readout that is visible to the naked eye. This isothermal DNA-detection scheme can be performed at temperatures between 20–45 °C. As an example of the applicability of the approach, we isothermally (23 °C) detected DNA from a parasite causing visceral leishmaniasis that was spiked into buffer and resulted in a sensitivity of at least 1 zeptomole. For proof of principle, DNA spiked into blood was coupled to the CRISPR-dCas9-based detection scheme yielding a colorimetric readout visible to the naked eye. Given the versatility of the guide-RNA programmability of targets, we envision that this DNA detection scheme can be adapted to detect any DNA with minimal means, which facilitates applications such as point-of-care diagnostics in resource-limited settings.

## Abstract 2

Nucleic-acid detection is crucial for basic research as well as for applications in medicine such as diagnostics.

## Graphical abstract

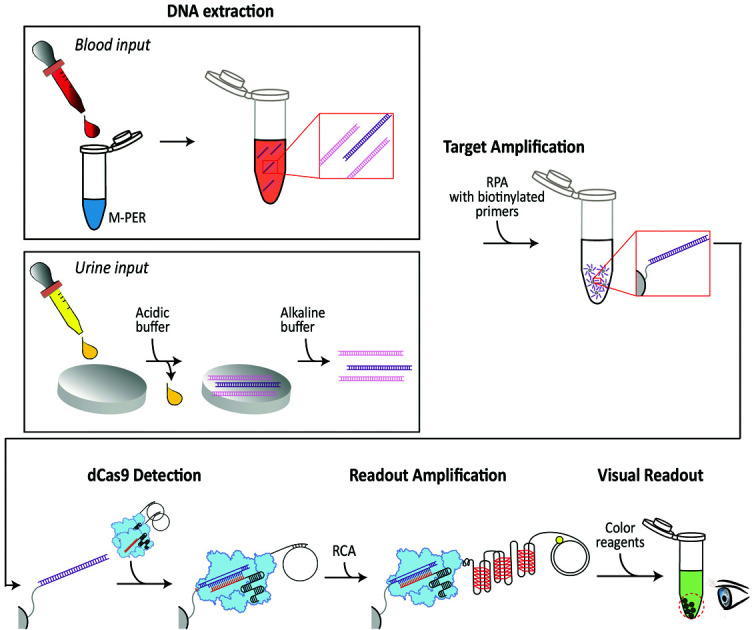

Effective treatment and containment of infectious diseases requires accurate diagnostics.^1,2^ Diagnostics are either based on direct detection by visualizing the pathogen using microscopy, or based on indirect detection such as immunological (antigen/antibody) or molecular (nucleic acid) detection.^3^ Though direct detection is accurate and the current gold standard for infectious disease diagnosis, it is laborious and has a slow turnaround time for results (∼1 week). Additionally, it requires trained personnel and sophisticated equipment. Thus, indirect detection is a preferred choice for disease diagnosis. Serological tests that detect antibodies from the infected person provide a quick and simple screening tool, but their efficacy differs between individuals and populations, due to inherently different immunological responses.^4^ Immunological tests furthermore cannot be used to test the efficacy of treatment (test-of-cure) or re-infection (relapse), due to persisting antibodies after treatment.^5,21^ Molecular nucleic-acid detection^3^ is beneficial for such diagnostic needs. DNA detection is vital in many biosensing applications in medical diagnostics,^6^ antimicrobial resistance testing,^7^ forensics,^8^ and many other areas.^9–14^ Molecular methods for DNA detection include quantitative polymerase chain reaction (qPCR),^15^ molecular hybridization techniques,^15^ DNA fluorescence *in situ* hybridization,^16^ as well as DNA sequencing using platforms such as next-generation^15^ or nanopore^17,18^ sequencing. However, in resource-limited settings, the use of such methods for point-of-care (PoC) diagnosis is severely hampered because it relies on expensive instruments (*e.g.* a thermal cycler for PCR), trained personnel to operate it, and a stable source of electricity,^19–21^ all of which often lack in many endemic regions where infectious diseases like neglected tropical diseases (NTDs) thrive.^22^ Therefore, alternative rapid, sensitive and specific confirmatory PoC diagnostic tests are urgently needed.^23^

Recent additions to this broad spectrum of DNA-detection methods include the use of DNA-binding proteins, such as Clustered Regularly Interspaced Short Palindromic Repeats (CRISPR) systems and their associated Cas proteins that have been adapted from bacterial immune systems.^24^ Indeed, programmable nucleic-acid-binding protein systems like CRISPR-Cas systems have shown promise for DNA-based PoC diagnostics. Notable examples include detection platforms called CRISDA,^25^ Cas9 detection for Zika virus,^26^ DNA Endonuclease-Targeted CRISPR Trans Reporter (DETECTR),^27^ and Specific High Sensitivity Enzymatic Reporter UnLOCKING (SHERLOCK).^28^ Although SHERLOCK and DETECTR have achieved impressive attomolar sensitivities,^29^ they still require sophisticated laboratory equipment as several of the handling steps are restricted to multiple incubations at different elevated temperatures. Furthermore, one of the greatest challenges for PoC diagnosis in resource-limiting settings is sample preparation,^30–32^ which adds additional handling steps and infrastructural requirements. The SHERLOCK detection platform,^28^ for example, has been coupled to a sample preparation technique known as HUDSON, which relies on heating the samples to 50 °C to inactivate nucleases, and to 90 °C to inactivate viruses, before detection by CRISPR-Cas13a^31^ can be initiated. A DNA-based PoC diagnostic method that is fully functional at room temperature has so far not been achieved, while there is a clear need for new diagnostic tools that enable minimally trained users to probe for diseases with minimal handling and resources.^33^

All CRISPR-Cas-based diagnostic platforms boost their sensitivity by including an initial nucleic acid amplification reaction.^29^ Loop-mediated isothermal amplification (LAMP), which operates at a constant but elevated temperature of 65 °C, is extensively used for such PoC diagnostics, and is commercially available.^20,29,34–36^ Other detection platforms use Recombinase Polymerase Amplification (RPA), a prominent example of an isothermal amplification technique that does not require heating, and is available as a commercial kit.^37^ Rolling Circle Amplification (RCA) has also been used for initial amplification for detection of micro-RNA in urine at 37 °C using a Cas9 mutant (dCas9) that specifically binds dsDNA but does not cleave it.^38^

Here, we present an isothermal DNA-detection scheme that detects DNA from a pathogen in human samples through a colorimetric readout that is visible to the naked eye (Fig. 1). We combine this DNA-detection scheme with two instrument-free DNA-extraction procedures, one for blood input and one for urine input,^39,40^ whereupon the extracted DNA is isothermally amplified by RPA. The RPA reaction is performed with a biotinylated primer, which facilitates the binding of the amplified DNA to streptavidin beads in a tube. The immobilized DNA is bound by a dCas9 protein that is functionalized with an oligonucleotide which is hybridized to a single stranded DNA (ssDNA) circle. Upon specific recognition of the target DNA in the sample by the dCas9-oligonucleotide complex, the circular ssDNA primes a subsequent RCA reaction. The circular RCA template encodes enzymatic G-quadruplexes that produce the final colorimetric readout. Below, we describe the successful operation of all these steps, and we specify the favorable sensitivity and temperature ranges where the method operates – demonstrating its potential for PoC diagnostics in resource-limited settings.

**Fig. 1:**
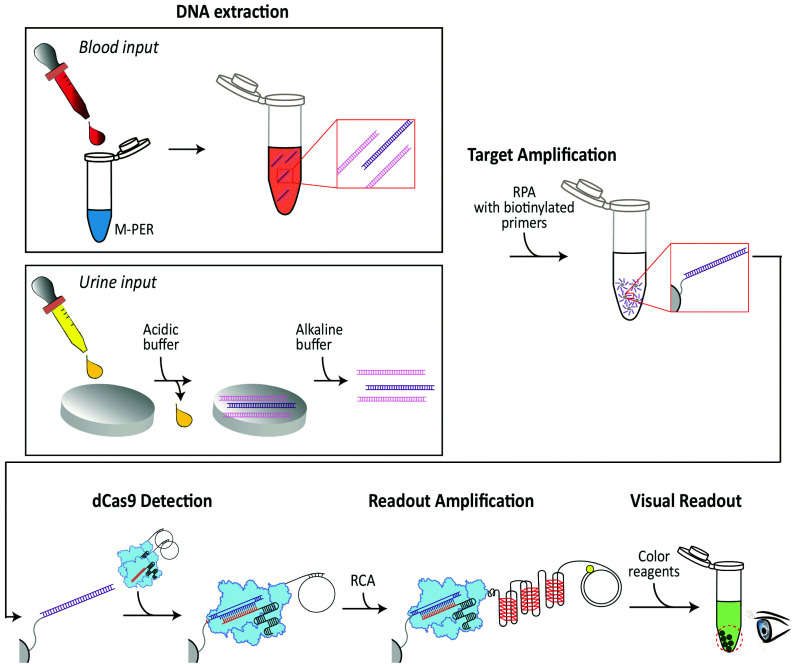
Schematic of the DNA detection. DNA extraction: (Blood input) A blood sample is added to mammalian extraction buffer (M-PER) resulting in lysis of blood cells and release of intracellular DNA. (Urine input) A urine sample (acidic) is administered onto chitosan-functionalized paper discs, resulting in entrapping of total DNA (target DNA (purple) in a background of non-target DNA (pink), which is subsequently released upon washing with an alkaline buffer. Target Amplification: Only target DNA (purple) is isothermally amplified using RPA with a biotinylated primer. Subsequently, the target DNA is immobilized to streptavidin-coupled beads *via* biotin-streptavidin interactions. dCas9 detection: The RPA-amplified immobilized target DNA is recognized by CRISPR-dCas9 (light blue) that has a single-stranded DNA (ssDNA) circle attached. Anything that is not bound to the beads is washed away. Readout amplification: The circular ssDNA is used to prime an RCA reaction. The resulting RCA product consists of tandem repeats of G-quadruplexes. Each of these G-quadruplexes picks up a heme group and a colorimetric readout is produced where a visible color appears on the beads in the tube when the target DNA sequence is present.

For proof-of-principle, we demonstrate the applicability of the DNA detection scheme for the diagnosis of the NTD leishmaniasis.^41^ Visceral leishmaniasis (VL), also known as Kala-azar, is caused by protozoan parasites of the genus *Leishmania* (in particular *L. donovani* and *L.* infantum) that affect the visceral organs (liver, spleen and lymph nodes). VL is curable, but it persists as a fatal disease as it is often left undiagnosed and untreated.^42^ Current VL rapid diagnostic tests are serological and sub-optimal,^43^ indicating a need for PoC development for DNA-based diagnostics in resource-limited settings. To diagnose VL using our DNA detection scheme, a highly conserved multicopy region (∼10 000 copies/parasite) was identified in the *Leishmania* kinetoplast minicircle DNA, and its presence was validated within a patient's blood and urine sample. We thus demonstrate the presence of VL DNA in a buffer sample with a colorimetric readout that is visible to the naked eye. One zeptomole of VL DNA led to an observable color change in the tube, corresponding to approximately 600 molecules or only 6% of the DNA of one parasite. We also demonstrate the presence of VL DNA in a blood sample with a colorimetric readout that is visible to the naked eye. Notably, all steps in this detection scheme were performed at room temperature (23 °C). We anticipate that this DNA detection scheme will be broadly applicable for many other diseases and a wide range of biosensing applications.

## Results

### Target DNA in biological samples can be detected sensitively, fast, and across a wide range of temperatures

**Fig. 2:**
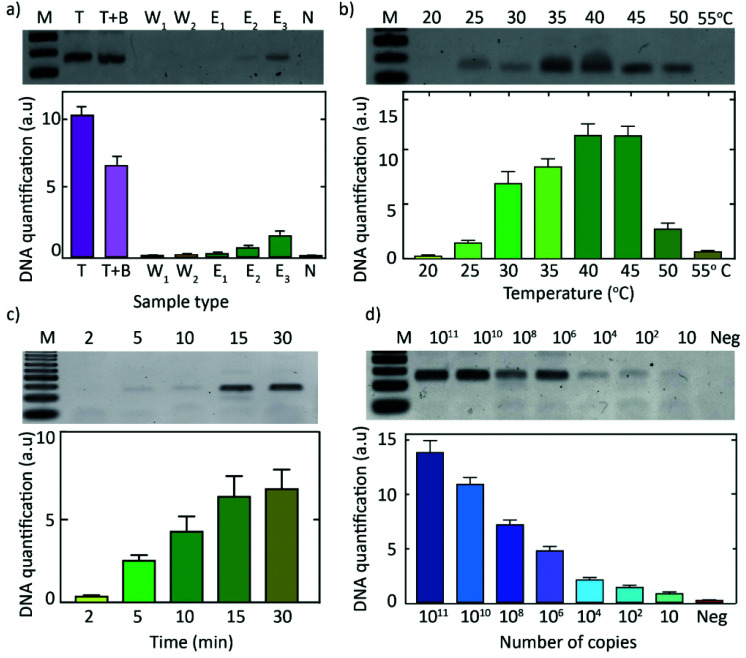
Chitosan-mediated DNA extraction and subsequent isothermal DNA amplification. (a) pH-based chitosan-mediated DNA extraction. Lane 1 (M): 50 bp DNA ladder showing 50, 100, 150 bp oligomers. Lane 2–9 show the 115 bp DNA band. Lane 2 (target, T): PCR-purified target (10^12^ copies). Lane 3: PCR-purified target in MES buffer, T + B (pH 5.0, 10 μL) that was added onto a chitosan-functionalized membrane. Lanes 4 and 5 (W1, W2): result of two separate washes with MES buffer (pH 5.0, 20 μL each) to remove unbound DNA. Lanes 6–8 (Elutes 1–3): 3 subsequent elution steps with Tris buffer (pH 8.0, 20 μL each) to extract the bound DNA. Lane 9 (neg): no-target control, *i.e.*, MES buffer (pH 5.0, 10 μL) that was added onto chitosan-functionalized membrane and eluted with Tris buffer (pH 8.0, 20 μl). (b) Effect of the operating temperature on the RPA reaction, where DNA from the third elution step (E3 from Fig. 2a) was followed by isothermal amplification (RPA) for 30 min at different temperatures from 20 to 55 °C. (c) Effect of the duration of the RPA reaction, where elute 3 from Fig. 2a was followed by RPA for different durations, *i.e.*, 2–30 min at 39 °C. (d) Sensitivity of the RPA reaction: RPA reaction results for different input concentrations of target DNA, ranging from 10^11^ to 10 copies, that were added unto the chitosan-functionalized membrane for DNA extraction followed by RPA at 39 °C for 30 min. All gels are examples of a single experiment, while data in the bottom quantifications are plotted for *n* ≥ 3.

The first steps in our DNA-detection scheme (Fig. 1) involve the extraction and amplification of target DNA. To lyse the blood cells in the sample, we utilized a commercially available Mammalian Protein Extraction Reagent (M-PER) which was used as a mild detergent lysis that was shown to be compatible with the subsequent RPA reaction. To isolate DNA from urine samples, however, we utilized a pH-based chitosan-mediated DNA-extraction procedure^44^ wherein, under acidic conditions, DNA is electrostatically adsorbed onto chitosan-functionalized paper discs (ESI Fig. 1†), and subsequently eluted with an alkaline buffer wash. To test the chitosan-mediated DNA-extraction procedure, we added target DNA to an acidic buffer (MES buffer, pH 5) or a biological liquid (urine, adjusted to pH 5). Fluid (buffer or urine) was spiked with target DNA and administered onto chitosan-functionalized paper discs, washed with the acidic buffer (MES buffer, pH 5) to remove unbound constituents, and subsequently the bound DNA was eluted with alkaline buffer (Tris buffer, pH 8). Washes and eluates were analyzed using gel electrophoresis. As can be seen from Fig. 2a, DNA was successfully bound to the chitosan–functionalized membrane, while it could be eluted with three subsequent washes with an alkaline buffer (Tris pH 8.0).

The eluted DNA was used as a template for a downstream RPA reaction. The rehydration buffer from a commercial RPA kit (TwistDx) was used as the alkaline buffer for the elution of the DNA that was adsorbed onto the chitosan-functionalized paper discs. Owing to the small size of the chitosan-functionalised paper-discs (∼6 mm), not all the target DNA was bound to the membrane and hence, the extraction was below 50% (Fig. 2a). Importantly, however, due to the high sensitivity of the RPA reaction, the target was amplified to the saturation levels irrespective of an initial loss of target DNA that did not bind to the chitosan-functionalized paper discs.

The DNA extraction and the subsequent isothermal–amplification steps (*i.e.* RPA) were functional at temperatures ranging from 25 °C to 50 °C (Fig. 2b). The RPA assay produced a read-out within 5 minutes (Fig. 2c). The assay has an excellent sensitivity and can identify amounts as small as 10 target DNA copies, and an upper limit of at least 10^11^ target DNA copies in the volume corresponding to a blood prick (10 μL) (Fig. 2d). The pH-based chitosan-mediated DNA-extraction approach followed by the RPA reaction exhibited the same detection limit as that of the RPA reaction alone (ESI Fig. 2†), indicating that the reactions were compatible and that the chitosan approach did not hinder the amplification efficiency.

Subsequently, biotinylated primers were used in the RPA reaction to facilitate immobilization of the amplified target DNA onto streptavidin-coated beads (Fig. 1, top right), thus allowing to wash off unwanted reagents. This wash step resulted in a clean amplified target DNA for the subsequent dCas9-based recognition. Notably, the primers could either be immobilized to the streptavidin-coated beads before or during the RPA reaction (ESI Fig. 3†).

### CRISPR-dCas9 on target DNA can be bound, amplified, and visualized with a colorimetric readout

**Fig. 3:**
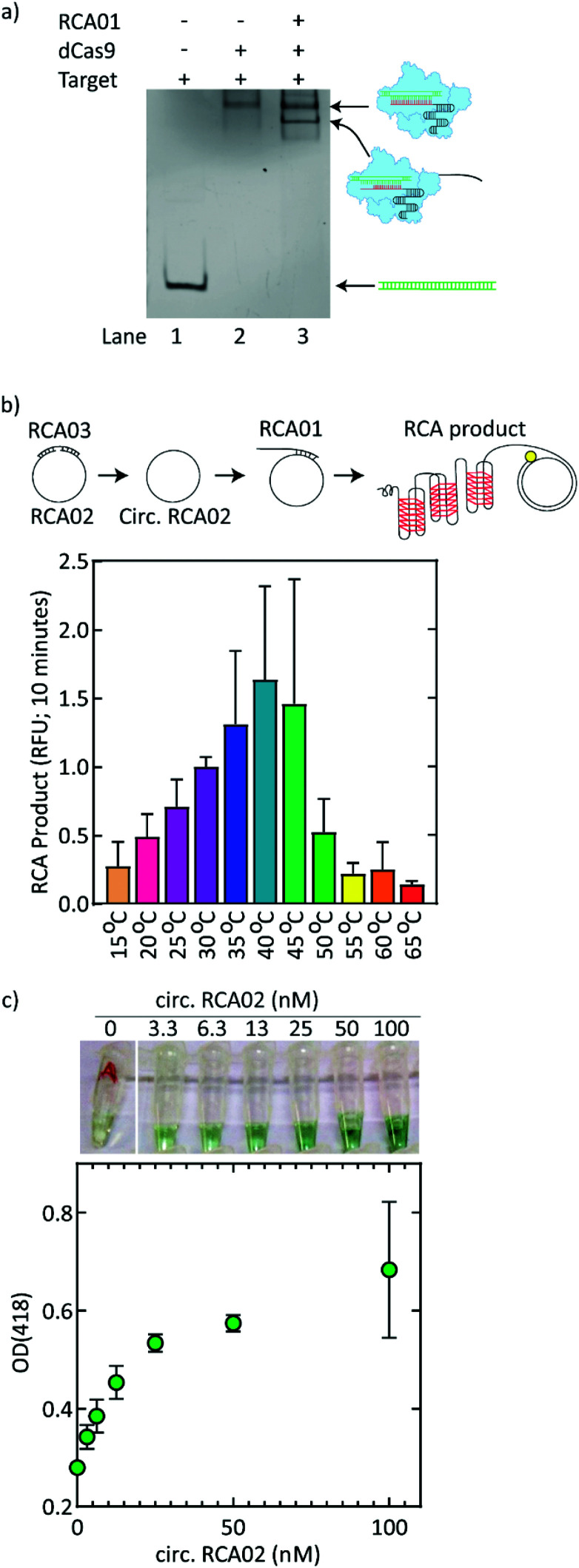
Detection of target DNA using CRISPR-dCas9 and RCA. (a) Representative shift assay with target DNA incubated either alone (lane 1), with sgRNA-dCas9 (lane 2), or with sgRNA-dCas9-RCA01 (lane 3). The lower-mobility band in lane 3, represents some dCas9 proteins without RCA01 (see also ESI Fig. 5†). (b) Fluorescence *versus* time for RCA reactions at temperatures between 15 °C and 65 °C. The top panel illustrates the formation of the RCA product. RCA02 is ligated with RCA03 (bridge). The resulting circularized RCA02 is exonuclease digested to remove remaining single-stranded RCA03. RCA01 primes the RCA reaction to yield a RCA product. The bottom panel shows the fluorescence readout (relative fluorescent units; RFU) after 10 minutes of the RCA reaction *versus* temperature (*n* = 3). (c) OD_418_*versus* RCA02 circle concentration (nM) for RCA reactions at room temperature (23 °C) after 24 hours (*n* = 3). Representative pictures of the resulting color are shown above the graph.

In a next step, we employed the high sequence-specificity of the CRISPR-dCas9 system to enhance the specificity of the targeting of pathogenic DNA. To subsequently couple the DNA detection by CRISPR-dCas9 to the colorimetric readout, the dCas9 protein was covalently linked to a DNA oligonucleotide (named RCA01) which served as a primer for an RCA reaction (Fig. 3a). To demonstrate that the dCas9-RCA01 complex efficiently binds to the RPA-amplified target DNA, we performed an electrophoretic mobility shift assay (EMSA). sgRNA was preincubated with the dCas9-RCA01 complex to form a sgRNA-dCas9-RCA01 complex, which was then incubated with the target DNA. The EMSA showed that the sgRNA-dCas9-RCA01 complex bound to the target DNA (lanes 2 and 3 in Fig. 3a), as seen from a shift with respect to the unbound target DNA (lane 1 in Fig. 3a). An excess of dCas9-RCA01 over target DNA was used in the reaction to ensure that all target DNA was bound by the dCas9-RCA01 (ESI Fig. 4†).

In a second amplification step, RCA01 was hybridized to a circular RCA template (circularized RCA02) to facilitate the RCA reaction. RCA was thus used to yield a long repetitive ssDNA molecule that contained tandem repeats of G-quadruplexes, which in turn facilitate a colorimetric readout. The circular ssDNA is made using a 109-mer linear oligonucleotide template (named RCA02) with a sequence that encodes four tandem repeats of the G-quadruplex structure, plus a 20-mer linear oligonucleotide (named RCA03) that serves as a bridging oligonucleotide that hybridizes to the two ends of RCA02 to facilitate ligation of these ends by T4 ligase to covalently close the circular template (Fig. 3b, top panel). This template design facilitated efficient circularization by ligation (ESI Fig. 6†). The resulting circularized RCA02 sample was exonuclease digested to remove remaining single-stranded RCA03. The RCA01 oligonucleotide primed the RCA reaction and yielded a massive RCA product (with a linear length of 200 nm to 5 μm)^45,46^ that contains a repetitive sequence complementary to that of the RCA02 circle. Fig. 3 depicts the results of the RCA reaction. DNA production was monitored from the SYBR Green I fluorescence signal that was produced over 60 minutes. The RCA reaction was examined in a range of temperatures. Our template design allows RCA to be conducted at all temperatures between 15 °C and 60 °C, while it is optimal for temperatures of 25–40 °C (Fig. 3b and ESI Fig. 7†).

The resultant RCA product encodes for G-quadruplexes which have peroxidase activity when they are in complex with hemin.^47^ Upon the addition of hemin, ABTS^2−^, and hydrogen peroxide to the final RCA product which contained the G-quadruplex DNA due to the RCA reaction, it thus changed in color over time, as the hemin would bind to the G-quadruplexes and facilitate the conversion of ABTS^2−^ into the colored ABTS˙^−^ in the presence of the hydrogen peroxide. This change in color was visible to the naked eye. Indeed, following extensive RCA for 24 h at room temperature (23 °C), reagents for the color reaction (hemin, ABTS^2−^, and hydrogen peroxide) were added, and the resulting color change could be measured using the optical density OD_418_ as well as observed by the naked eye (Fig. 3c). Alternative solutions containing more stable hydrogen peroxides were examined and compared (ESI Fig. 8†). Notably, the RCA experiment shown in Fig. 3c was conducted at room temperature in the absence of any equipment, exemplifying the broad applicability of the assay.

### DNA detection of the NTD pathogen causing visceral leishmaniasis

**Fig. 4:**
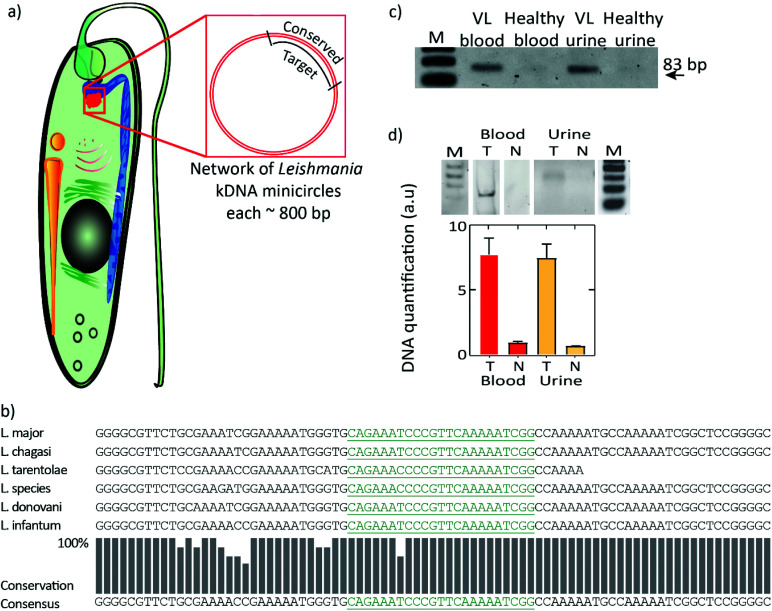
Target selection for diagnosis of visceral leishmaniasis. (a) Schematic of a *Leishmania* parasite. Inset: Kinetoplast minicircle DNA that was identified (∼10 000 copies per cell) as a target. (b) Target sequence identification: A multiple-alignment tool was used to identify a consensus region across the pan-*leishmania* genus that could serve as a potential target. Black letters denote the 115 bp largely conserved sequence. Multiple iterations yielded putative targets within the kinetoplast minicircle DNA for recognition of various strains of leishmaniasis, *viz.*, *L. major*, *L. chagasi*, *L. infantum*, *L. donovani*, and *L. tarentolae*. The green letters denote a conserved 23-mer sequence that was selected as the final target, as it can serve as a CRISPR-dCas9 binding sequence, and as it has no homology with the human genome and non-VL pathogenic genomes. (c) PCR showing the presence of the VL target in patient blood and urine, Lane 1 (M): 50 bp DNA ladder showing 50, 100, 150 bp oligomers. Lane 2: PCR amplified target (83 bp) from a positive VL patient's blood sample. Lane 3: healthy blood (DNA extracted using kit from 500 μl of blood). Lane 4: PCR amplified target (83 bp) from a positive VL patient's urine sample, lane 5: healthy urine (circulating cell free DNA extracted using kit from 13 ml of urine). (d) DNA was extracted using two different instrument-free methods for urine and blood samples. For blood samples spiked with target (2 × 10^11^ molecules in 10 μl): M-PER mediated DNA extraction and subsequent RPA at 39 °C for 30 min (analysed by polyacrylamide gel electrophoresis). For urine samples (*n* = 3) spiked with target (2 × 10^11^ molecules in 10 μl): chitosan-mediated DNA extraction and subsequent RPA at 39 °C for 30 min (analysed by agarose gel electrophoresis). T denotes sample spiked with target DNA, and N denotes negative control. All gels are examples of a single experiment, while data in the bottom quantifications are plotted for *n* ≥ 3.

While this CRISPR-dCas9-based DNA detection scheme may find a wide range of applications, we here show one example that is geared at PoC testing in resource-limited settings of parasitic DNA from a VL patient. We selected kinetoplast minicircle DNA as the target, as a single parasite contains about 10 000 copies of this minicircle DNA^48^ (Fig. 4a). To identify a specific consensus region across all the *Leishmania* species within the ∼800 bp kinetoplast minicircle, a multiple sequence alignment tool called T-coffee was used.^49^ The identified consensus sequences were further analysed for homologies (sequence similarities) against other pathogen's genomes, including *Trypanosoma* and *Plasmodium* species which may co-exist in VL-endemic regions, and against the human genome using BLAST.^49^ Unique DNA sequences with no sequence similarities to other related pathogens genomes or human genome were chosen as targets for CRISPR-dCas9 based DNA detection scheme for VL-infection.

A potential target sequence of 115 bp was identified that contained the dCas9 protospacer adjacent motif (PAM) recognition site (NGG for *Streptococcus pyogenes* Cas9). The green letters denote a conserved 23-mer sequence that was selected as the final target, as it can serve as a CRISPR-dCas9 binding sequence while it has no homology with the human genome and non-VL pathogenic genomes (Fig. 4b and ESI Fig. 9†). To verify if the target gene was present in patient's samples such as blood and urine, PCR was performed on DNA extracted from a VL patient's blood and urine sample, as well as from a blood and urine samples from healthy humans as negative controls, using a set of primers that yielded an 83 bp DNA product that is present within the 115 bp consensus target sequence (Fig. 4c). The results confirmed the presence of the target DNA in the VL patient blood and urine samples. Two DNA extraction procedures were included in this study, *i.e.* M-PER mediated DNA extraction from blood and chitosan-mediated DNA extraction from urine. In both cases, samples were spiked with target DNA (2 × 10^11^ VL target molecules in 10 μL of sample), followed by RPA (Fig. 4d). The extraction-RPA procedure was found to work directly from crude blood and urine samples.

**Fig. 5:**
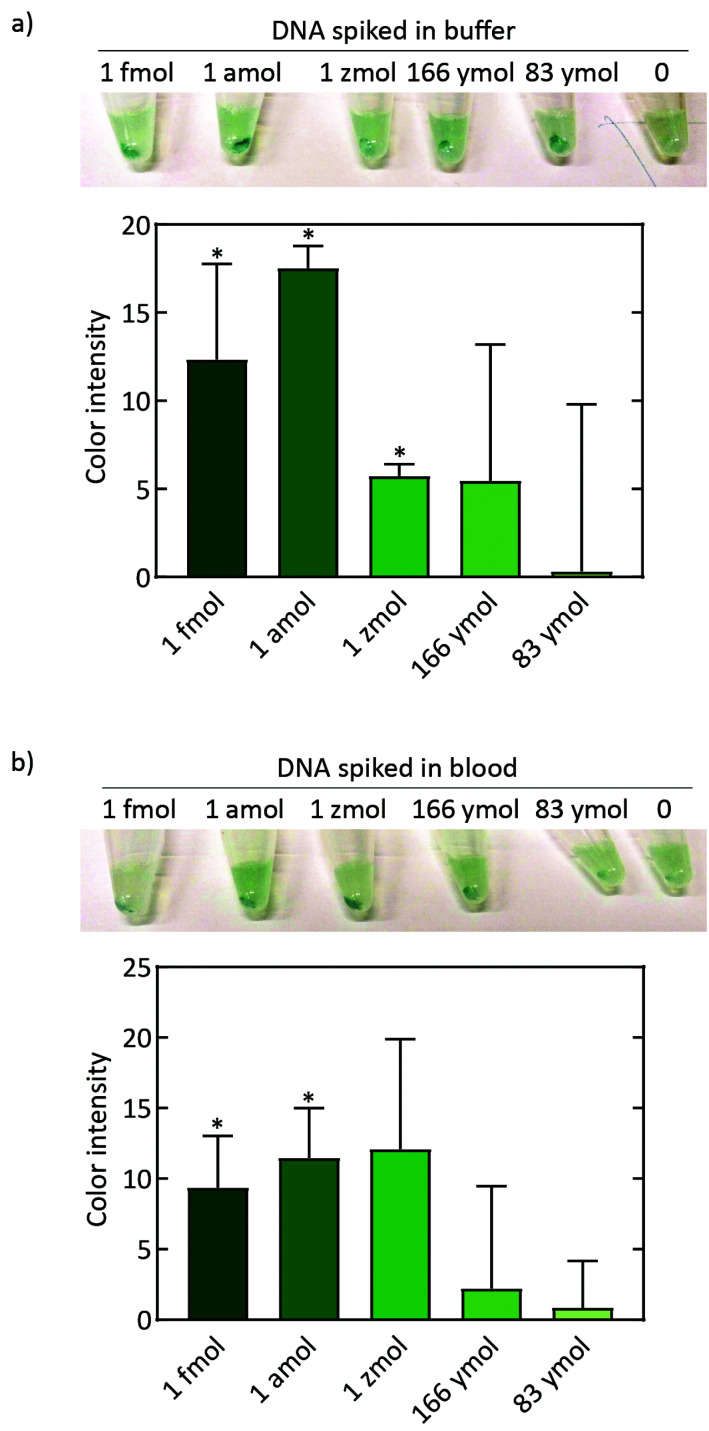
Complete workflow of the DNA-based detection scheme. (a) Sensitivity of the assay using naked DNA. RPA reactions were spiked with DNA from 0 to 1 fmol of DNA molecules, followed by the remaining steps in the DNA-detection scheme. The top panel shows an image of the final reaction after the colorimetric readout. The bar-chart below the image shows the color intensity of the precipitated beads from images of the tubes in three independent experiments, where the background has been subtracted (*n* = 3). Color intensity of the beads was measured using ImageJ. (b) Sensitivity of the full assay using simulated patient samples from DNA extraction with M-PER to the final color reaction in the tubes. The top panel shows an image of the test tubes after the colorimetric readout. The bar-chart below shows quantification of the color intensity of the precipitated beads in the tubes in three independent experiments, where the background has been subtracted (*n* = 3). Color intensity of the beads was measured using ImageJ. Asterisk illustrate that the sample test significantly different from sample without target DNA.

Furthermore, the ability of the DNA detection scheme to detect target DNA was examined using a titration of VL target sequence (Fig. 5). VL target DNA was added to the RPA reaction followed by immobilization on streptavidin-coated beads, subsequent dCas9 detection, and isothermal amplification by RCA (using the circular template RCA04), all at room temperature (23 °C), before reagents for the colorimetric readout were added and resulting color was observed with the naked eye (Fig. 5a, top panel). The color intensity of the beads was quantified in images of the tubes, and the color intensity in the tube with zero target molecules was subtracted to allow comparison across experiments (Fig. 5a, graph; see also ESI Fig. 10†). Using naked DNA spiked into buffer, we thus reached a sensitivity of at least 1 zeptomole (∼600 molecules).

Finally, we examined the detection limit of the full detection scheme using simulated patient samples. For proof of principle, we combined the full DNA detection scheme with M-PER extraction from a blood sample spiked with our target DNA sequence (Fig. 5b). The color intensity of the beads was observed in the tubes (Fig. 5b, top panel) and quantified as described above (Fig. 5b, graph). With these blood samples, we observed a lower limit of detection that lies somewhere in between 1 zeptomole and 1 attomole.

## Discussion and conclusion

We developed a DNA detection scheme that is broadly applicable for biosensing applications such as diagnostics. We demonstrated that target DNA can be isolated from biological samples sensitively, specifically, and across a broad temperature range. Target DNA was amplified, bound by CRISPR-dCas9, amplified further, and visualized with a colorimetric readout that was visible with a naked eye. The ability of the dCas9-RCA01 complex to bind to the RPA-amplified target DNA ensures the specificity and robustness of the direct DNA-detection scheme. The combination of RPA, CRISPR-Cas9, and RCA amplification led to a very high selectivity and sensitivity. The visible readout to the naked eye and functionality at room temperature throughout all extraction and detection steps confer specific advantages for facile application.

With these favorable characteristics, *viz*., room-temperature operation without the need of electricity, we developed a DNA-detection scheme that is suitable for disease diagnosis in resource-limiting settings. Since we aim to apply this DNA detection scheme as an instrument-free diagnostic test, we first utilized a pH-based chitosan-mediated DNA-extraction for urine samples and a M-PER mediated DNA-extraction from blood samples. Our results demonstrated that these DNA-extraction methods were fully compatible with the downstream RPA reaction, and suggest that it can be applied to detect even a single parasite in a pin-prick of blood (∼10 μL) or fewer than 10 copies of circulating cell-free DNA in a urine sample from a patient. Since ‘room temperature’ can differ substantially in the global South where many NTDs remain endemic,^50^ it is advantageous that the isothermal reactions used in this detection scheme perform efficiently across a wide range of temperatures up to 40 °C. Notably, our isothermal DNA extraction method does not require any temperatures beyond room temperature, which contrasts other DNA detection platforms for resource-limiting settings, such as HUDSON which relies on heating the samples to extract DNA before detection by CRISPR-Cas.^31^

Despite these positive conditions, it is useful to discuss potential pitfalls of our approach, mostly related to the many handling steps that are required in this DNA-based detection scheme from DNA extraction to visual read-out. Since the assay includes an ultra-sensitive RPA reaction, false positive RPA results are a well-acknowledged potential pitfall. RPA is known to amplify aerosols of target DNA that can contaminate the RPA reaction, and such aerosols may persist from previous days. Therefore, RPA reactions must be handled with caution. In our experiments, sample-preparation and DNA-extraction steps were always performed in a pre-amplification room to separate the downstream steps of the assay. These cautionary measures are compliant with Good Laboratory Practice measures when performing molecular amplification assays.^51^ False positives may in part also result from improper washes during manual pipetting, *e.g.* where residual dCas9-RCA01 complex remains after insufficient washing, which in turn can lead to a false positive signal. Finally, the reagents (particularly the enzymes), were refrigerated throughout this proof-of-principle study. Thus, lyophilization of the reagents will need to be validated in follow-up studies on developing a packaged diagnostic device. Furthermore, since access to patient samples was limited in our study, a next step would be to apply the complete DNA-detection scheme to a broad range of patient samples from different populations to further validate the applicability of the scheme.

Due to the ease of programmability of the CRISPR-dCas9 system, our DNA detection scheme can in principle be programmed to detect any pathogenic DNA, genetic variants (SNPs, insertions, deletions), and antimicrobial resistant strains, as well as be used in other biosensing applications such as forensics and genotyping. We envision that the diagnostic scheme presented in this study can be packaged into a closed automated microfluidic device with sample-in answer-out capabilities for testing blood or urine samples as a field- or home-deployable diagnostic test using lyophilized reagents. Such a closed system will prevent non-specific amplification and/or amplification of aerosols of target DNA. Furthermore, automated wash steps may in future work be accommodated in a microfluidic device, *i.e.*, by releasing washings buffers from dedicated reservoirs in the device (ESI Fig. 11†). In addition to providing a valuable tool in epidemics, such a test will be of great use for diagnosis of the persistent NTDs currently affecting more than 1 billion people worldwide.^1^ For most NTDs, diagnostic tests are ineffective due to a lack of resources, which calls for field-deployable PoC diagnostics with a sensitive, specific, user-friendly, rapid, robust, affordable and equipment-free DNA-detection scheme.^52^ We envision that our confirmatory DNA-detection scheme can potentially replace cumbersome culturing and microscopy-based diagnostic procedures by providing accurate real-time results at the point-of-care. Since our DNA-detection scheme can function independently of the patients’ immune response, it can be applied to all ethnic populations and present a test-of-cure and test-of-relapse of infections. Moreover, the cost of a diagnostic device based on our detection scheme can be expected to be low since similar CRISPR/Cas-based molecular diagnostic tests such as SHERLOCK and DETECTR cost <$0.1 per reaction for reagents, which is reasonable for low-resource applications.^21^

In conclusion, the detection scheme presented in this study is advantageous over other nucleic acid detecting methods for diagnostics in resource-limited settings, as it does not require electricity, advanced equipment, or expert personnel to operate, and it can be designed to very sensitively detect a broad range of DNA targets. We anticipate that this simple, specific, and sensitive diagnostic scheme can be readily applied to address the diagnostic PoC needs of various infectious diseases and thus help alleviate the global healthcare burden.

## Materials and methods

### Target selection and optimization

A multiple-alignment tool (T-coffee software, tcoffee.crg.cat) was used to identify a consensus region across the pan-*Leishmania* (*L*) genus that could serve as a potential target. Multiple iterations yielded putative targets within kinetoplast minicircle DNA for recognition of *L. major*, *L. chagasi*, *L. infantum*, *L. donovani. L. tarentolae* and *L. amazonensis*. The identified targets were further checked for homology with human or non-L disease-causing pathogen's sequences using BLAST. A sequence of 115 bp was identified that contained a 23-mer CRISPR-dCas9 target that had no homology with other genomes (see ESI Fig. 8†). The synthetic gene was cloned into pUC 57 plasmid (GenScript (Leiden, Netherlands) and transformed in *E. coli* top10 cells. DNA was extracted using a Qiagen plasmid midi kit and the synthetic target gene construct was obtained using standard Phusion DNA polymerase PCR employing primers pairs, synthetic *leishmania* target forward (FWD) primer and synthetic *leishmania* target reverse (REV) primer, employing the following protocol: 98 °C for 3 minutes followed by 30 cycles of [98 °C for 10 seconds, then 58 °C for 20 seconds, and 72 °C for 15 seconds], with a final hold of 72 °C for 8 minutes. PCR product (target DNA) was checked on 3% agarose gel and further cleaned using a NEB monarch kit. For target detection within VL patient's blood and urine samples, primer pairs (VL target FWD PCR primer and VL target REV PCR primer) were used for standard Phusion DNA polymerase PCR (same protocol as above). The template for PCR was obtained using the genomic DNA extraction kit (Qiagen, Europe) utilizing 500 μl of VL patient's blood, and circulating cell free DNA extraction kit (Qiagen, Europe) utilizing 13 ml of VL patient's urine.

### Chitosan-based DNA extraction

10 cm long Fusion-5 filter paper strips were plasma-cleaned (2–3 minutes) prior to overnight incubation in chitosan solution (0.05% w/v in 0.1% acetic acid, pH 6.0) at room temperature. Thereafter, chitosan-functionalized paper was washed three times with deionized water, dried at 60 °C for 1 hour and stored at room temperature. 10 mM MES buffer (pH 5.0) supplemented with target DNA (at different concentrations) was used to mimic patient's sample. To entrap the DNA, 10 μl of MES buffer with target DNA was added to 6 ± 0.5 mm sized paper-discs of chitosan-functionalized paper and incubated for 5 minutes. To elute the target DNA, 20 μL of 50 mM Tris(hydroxymethyl)aminomethane (Tris) (pH 7.9) or 20 μL of the rehydration buffer from the RPA kit was added directly unto the DNA entrapped chitosan-functionalized paper. The eluate was used as a template for the subsequent isothermal DNA amplification.

### M-PER mediated DNA extraction

40 μL blood spiked with target DNA was added to a tube containing 40 μL M-PER MAMMALIAN PROT EXTRACT (thermofisher). The liquid was mixed before 10 μL of the resulting solution was added to the RPA reaction mixture.

### Preparation of streptavidin coupled beads

High Capacity Streptavidin Agarose Resin (Thermofisher) was incubated at 37 °C for 1 hour while tumbling (Labnet, revolver) with 1% (w/v) BSA in 1× PBS (prepared from phosphate buffered saline tablets, Sigma Aldrich) in 1 : 4 volume ratios. 20 μL beads were used per reaction to immobilize RPA product from one RPA reaction tube (Twist Dx basic kit).

### Isothermal DNA amplification by RPA

Recombinase polymerase amplification was employed to obtain multiple copies of target DNA using the Twist Dx basic kit as per the manufacturer's instructions. The primer sequences, VL target FWD RPA and VL target REV RPA, were designed outside the CRISPR-dCas9 target region. Note that for Fig. 5, a biotinylated VL target FWD RPA primer and VL target REV RPA primer was used. Before adding the RPA mix to streptavidin beads, proteins in the RPA mix were removed using Protease K digestion. This was done by adding 50 μL of 2× proteinase k buffer (resulting in a final concentration of 30 mM Tris-Cl pH 8, 30 mM EDTA, 5% Tween 20, 0.5% Triton X-100, 800 mM GuHCl) and 40 units of Proteinase K (Proteinase K, Molecular Biology Grade, New England Biolabs) and incubating at 37 °C for 30 minutes followed by inactivation at 95 °C for 10 minutes for Fig. 5a; or at 23 °C for 30 minutes for Fig. 5b. The following RPA-Protease K solution was spiked into 500 μL of 1× PBS with complete inhibitors™ (Sigma Aldrich) and incubated with streptavidin coupled beads for 30 minutes at 23 °C while tumbling (Labnet, Revolver).

### sgRNA production

To make the single guide RNA (sgRNA), we first PCR amplified a dsDNA template, which contained the consensus sequence from a DNA plasmid (pgRNA-bacteria plasmid from Addgene), using a sgRNA FWD primer that contained a T7 promoter, and sgRNA REV primer. The following thermal cycling conditions were used to generate the PCR template: 98 °C for 3 minutes; 98 °C for 10 seconds; 65 °C for 20 seconds; 72 °C for 15 seconds; go to 98 °C for 10 seconds; 65 °C for 20 seconds; 72 °C for 15 seconds for 29 cycles and 72 °C for 8 minutes. The PCR template was verified using gel electrophoresis (1.5% agarose, 1× TBE buffer, 120 V for 90 minutes) and subsequently purified using the WizardSV Gel and PCR Clean-Up System (Promega) according to the manufacturer's instructions. sgRNA was then transcribed from the PCR template using the RiboMax™ Large Scale RNA Production Systems kit (Promega) according to the manufacturer's instructions. Following transcription, RNA products were purified using the RNeasy MinElute Cleanup Kit (Qiagen) according to the manufacturer's instructions. RNA quality was verified using gel electrophoresis (Mini-Protean TBE-Urea Precast Gels (Bio-Rad), 200 V for 30 minutes). Gels were visualized under UV light in a Biorad ChemiDOCT MP imaging system.

### sgRNA-dCas9-oligonucleotide complex assembly

To covalently attach an oligonucleotide to the dCas9 protein, an oligonucleotide sequence (RCA01) with a 5′ O_6_-benzylguanine (BG) group (Biomers) was incubated with the dCas9-Snap protein at 37 °C for 60 minutes. The dCas9-RCA01 complex was then purified using the AKTA pure chromatography system. We then assembled sgRNA, dCas9-RCA01, and DNA in a 1× NEBuffer 3.1 Reaction Buffer (New England Biolabs, 100 mM NaCl, 50 mM Tris-HCl, 10 mM MgCl_2_, 100ug mL^−1^ BSA, pH 7.9 at 25 °C) in a molar ratio of 100 : 10 : 1 (sgRNA/dCas9-RCA01/DNA). An excess ratio of dCas9-RCA01 was used to ensure maximum binding of the DNA to the protein. sgRNA was prepared by heating up to 95 °C for 10 minutes and slowly cooling down (1 °C every 4 minutes until a final temperature of 4 °C). sgRNA was then incubated with dCas9-RCA01 at 25 °C for 30 minutes. sgRNA-dCas9-RCA01 complexes were then incubated with DNA at 37 °C for 30 minutes. The binding affinity of the sgRNA-dCas9-RCA01 complexes to the DNA was verified using an Electrophoretic Mobility Shift Assay (EMSA) (10% 1× TBE-Precast Gels (Invitrogen), 90 V for 90 minutes). Gels were stained with Ethidium Bromide and visualized under UV light in a Biorad ChemiDOCT MP imaging system. For detection of naked DNA in Fig. 5, sgRNA, dCas9, RCA01 and circularized RCA02 were incubated in 1× NEBuffer 3.1 in concentration of 123.8 nM, 24.8 nM, 28.6 nM, and 19 nM respectively, in a total reaction volume of 105 μL. The reaction was incubated for 15 minutes at 23 °C. The streptavidin coupled beads were precipitated (500*g* 1 min) and the RPA-proteinase K solution was removed. Subsequently the beads were washed (1 mL PBS, followed by precipitation 500*g*, 1 min, and supernatant removed) twice, before the dCas9-sgRNA-circle mix was added to the tube containing the streptavidin coupled beads and tumbling at 23 °C for 30 minutes.

### Production of the circular RCA template and isothermal RCA reaction

The RCA template was produced using a template oligonucleotide RCA02. RCA02 was 5′-phosphorylated by T4 PolyNucleotide Kinase (PNK) for a final concentration of 1 μM and 0.1 units per μL, respectively. The 5′-phosphorylation reaction was performed for 60 minutes using 1x PNK buffer supplied by the manufacturer and 500 μM ATP. Following the 5′-phosphorylation, a primer oligonucleotide RCA03 was added for a final concentration of 3 μM, before all secondary structures in the DNA were disrupted by incubation at 95 °C for 10 minutes. The solution was allowed to cool to room temperature, before fresh ATP and T4 Ligase was added to the solution obtaining a concentration of 100 μM ATP and 0.4 units per μL T4 ligase, and the reaction proceeded for 16 hours at room temperature. The resulting circular template with primer was stored at 4 °C until usage (maximum 6 months). RCA was performed using a final concentration of 0.1 units per μL phi29 polymerase and 80 μM of nucleotides in 1× phi29 buffer, pH 7 and without DTT (33 mM Tris-acetate (pH 7 at 23 °C), 10 mM Mg-acetate, 66 mM K-acetate, 0.1% (v/v) Tween 20), and for Fig. 5a and b beads were precipitated (500*g* for 1 minute) before dCas9-sgRNA-circle mix was removed and the beads were washed twice with PBS before 100 μL of RCA mix was added and incubated at 23 °C for 16 hours while tumbling. In Fig. 3 RCA was performed at the indicated temperatures for 60 minutes, and RCA product generation was followed using 1× SyBr Green I and monitored fluorescence in QuantStudio™ 5.

### Colorimetric readout

To visualise the products of the RCA reaction with the naked eye, pH of the reaction was adjusted to pH 5 using 10 μL (citric acid-Na_2_HPO_4_) before adding 2.68 μL of 100 μM hemin (bovine hemin, Sigma Aldrich), 8.7 μL of 50 mM 2,2′-azino-bis (3-ethylbenzothiazoline-6-sulphonic acid (ABTS, Sigma Aldrich) and 8.7 μL of RefectoCil Oxidant 3%, GW cosmetics to the RCA reaction at room temperature. Thereafter, the color change was recorded with a digital camera. Quantification of the color intensity of the beads (and not the liquid in the tube) was performed using ImageJ. Quantified numbers were tested for significance (one-way, paired *T*-test) using GraphPad Prism software (version 9.3.0).

### Ethical statement

Anonymized clinical samples were derived from a pre-established Biobank at the Laboratory for Experimental Parasitology at the Academic Medical Centre (Amsterdam, The Netherlands) that were collected in accordance with the Dutch Medical Research involving Human Subjects Act and informed consent was obtained from human participants for the use of anonymized specimens according to the ‘no objection’ system.

## Conflicts of interest

There are no conflicts to declare.

## Supplementary Material

NR-014-D1NR06557B-s001
