# Supplementary material for: CRISPR-dCas9 based DNA detection scheme for diagnostics in resource-limited settings
Source: Nanoscale. 2022 Jan 19;14(5):1885–95. doi: 10.1039/d1nr06557b (PMC8812997; doi:10.1039/d1nr06557b)
Supplement: NR-014-D1NR06557B-s001 [file NR-014-D1NR06557B-s001.pdf]

Electronic Supplementary Material (ESI) for Nanoscale.
This journal is © The Royal Society of Chemistry 2022

Supplementary information

CRISPR-dCas9 based DNA detection scheme for diagnostics in resource-limited settings

Michel Bengtson*[,1], Mitasha Bharadwaj*[,1], Oskar Franch*[,1], Jaco van der Torre[1], Veronique Meerdink[1], Henk Schallig[2], and Cees Dekker[$,1]

[1]Department of Bionanoscience, Kavli Institute of Nanoscience Delft, Delft University of Technology, Delft, the Netherlands.

[2]Amsterdam University Medical Centers, Academic Medical Centre at the University of Amsterdam, Department of Medical Microbiology and Infection Prevention, Laboratory for Experimental Parasitology, Amsterdam institute for Infection and Immunity, Amsterdam, the Netherlands.

*Equal contribution

[$]Corresponding author

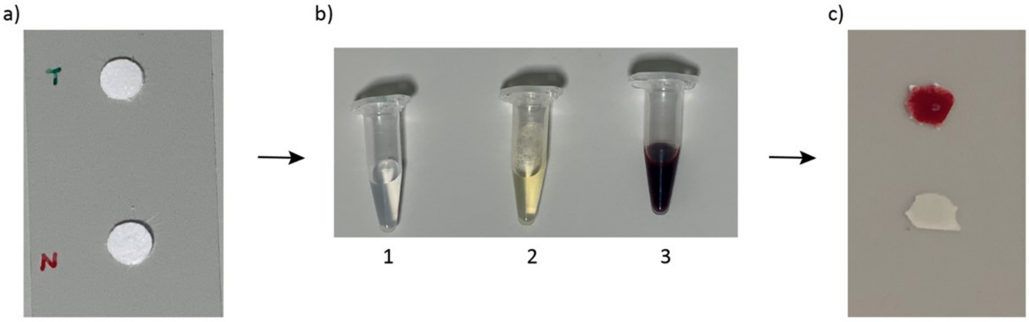

Supplementary Figure 1: pH-based chitosan-mediated scheme to extract target DNA.

a) Chitosan-functionlized fusion-5 paper discs (T=target; N=negative control). b) Target DNA is spiked into acidic buffer (1), urine (2), or healthy blood sample (3) and each fluid (pH 5.0.) is administered onto the chitosan-functionlized fusion-5 paper discs as seen in Figure S1a. Buffer, blood, or urine without target DNA are used as negative controls. c) The fusion-5 paper discs are placed inside the tubes (panel b) and washed with alkaline buffer (pH 8.0), to yield paper discs as seen in panel c. The DNA-containing elutes were further processed.

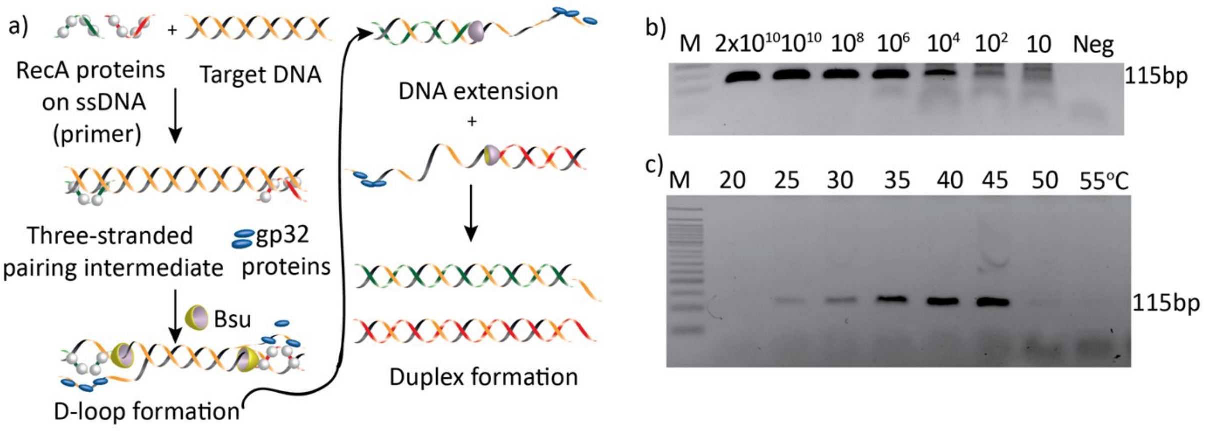

Supplementary Figure 2: Isothermal target DNA amplification.

a) Schematic of the working principle behind Recombinase Polymerase Amplification (RPA). Adapted from[52]. b) To determine the limit of detection for the RPA reaction, a titration of input target DNA from $2x10^{10}$ VL target molecules to 10 VL target molecules was amplified at 39°C for 30 mins. RPA can detect as few as 10 VL target molecules. c) To determine the operating temperature of the RPA reaction, RPA was then performed from 20°C to 55°C. RPA can amplify the target DNA across a broad temperature range from 25°C to 45°C.

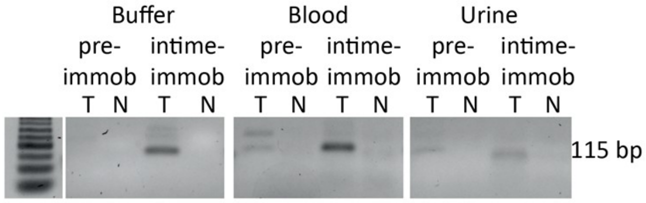

Supplementary Figure 3: Streptavidin-biotin immobilization of target DNA to a streptavidin-coated surface either before (‘pre-immob’) or during (‘intime immob’) the RPA reaction.

To determine if biotinylated-RPA primers should be immobilized to a streptavidin-coated surface before or during an RPA reaction, spiked-target DNA was extracted from buffer, blood or urine samples, and subsequently amplified by RPA with biotinylated-primers that were either immobilized to a streptavidin-coated surface before or during an RPA reaction. T denotes samples that contain spiked-target DNA. N denotes negative controls that do not contain spiked-target DNA. The results demonstrate that biotinylated-RPA primers should be immobilized to a streptavidin-coated surface during an RPA reaction for buffer samples. For both blood and urine samples, biotinylated-RPA primers could be immobilized to a streptavidin-coated surface before or during an RPA reaction, but more RPA amplicons were produced when biotinylated-RPA primers were immobilized during an RPA reaction. These results have implications for the final diagnostic device, and strongly suggest that biotinylated-RPA primers should be immobilized to a streptavidin-coated surface during an RPA reaction (“intime”).

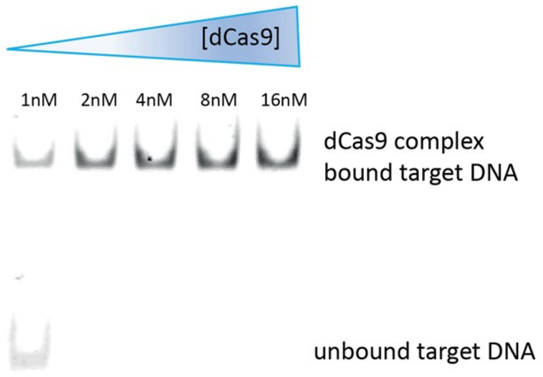

Supplementary Figure 4: dCas9 binding to dsDNA target.

EMSA using 1nM dsDNA target and increasing dCas9 concentrations (1nM-16nM). The upper band represents dCas9 bound to dsDNA while the lower band represent unbound dsDNA. An excess of dCas9 over target (minimum 2 times excess) is required to bind all the target DNA.

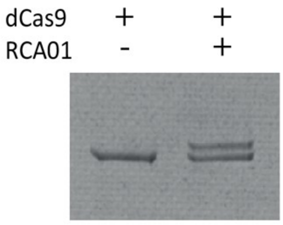

Supplementary Figure 5: Protein gel analysis.

dCas9 in the presence and absence of RCA01, lanes 1 and 2 respectively. The double bands in the presence of RCA01 shows that the covalent attachment of the oligonucleotide RCA01 is partially efficient. The upper band represents the proportion of dCas9 that is covalently attached to RCA01.

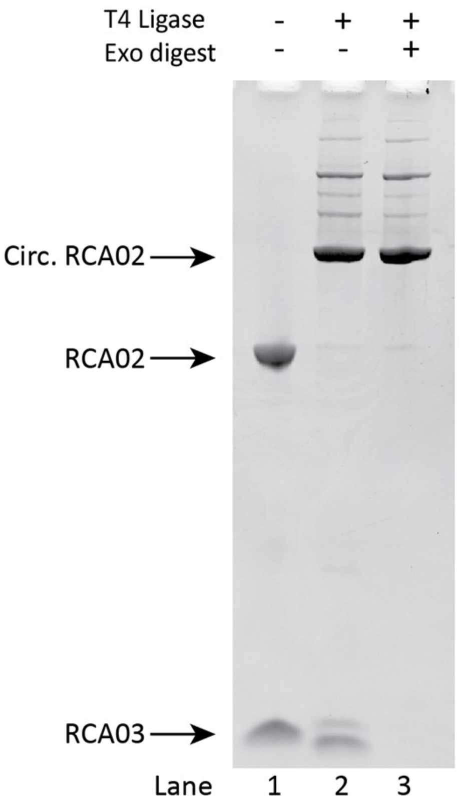

Supplementary Figure 6: Circular ligation efficiency.

To determine the ligation efficiency of the circularization technique used in this study, RCA02 and RCA03 were analysed in a denaturing gel. Lane 1 shows the input oligonucleotides RCA02 and RCA03 prior to any treatment. Lane 2 shows RCA02 and RCA03 after T4 ligation. Lane 3 shows RCA01 and RCA02 after T4 ligation and exonuclease treatment (“exo digest”). After the T4 ligation treatment, the RCA02 band disappeared while other low-mobility bands appeared (lane 2). After exonuclease treatment, the RCA03 bands disappeared, whereas the low-mobility bands remained (lane 3). These exonuclease-resistant low-mobility bands most likely represent larger circles of oligomers of RCA02. Most importantly, single circularized RCA02 was the major product (“circ. RCA02”).

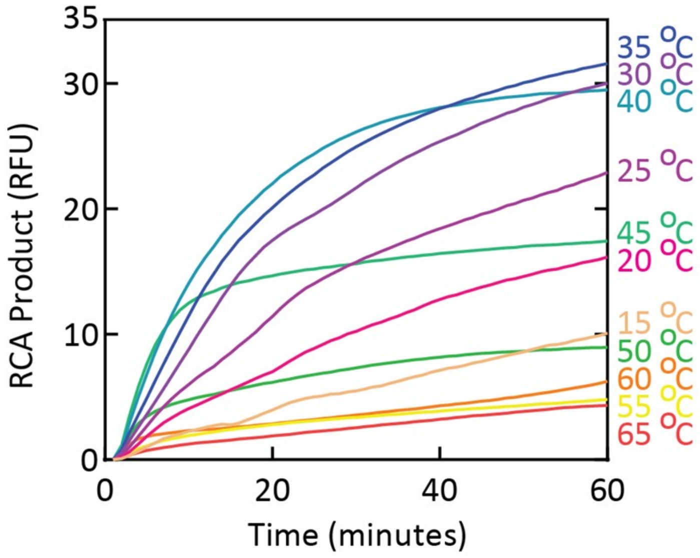

Supplementary Figure 7: Fluorescence versus time for RCA reactions at temperatures between 15°C and 65°C.

RCA product was monitored using SyBr Green I in a qPCR machine, as described for figure 3b.

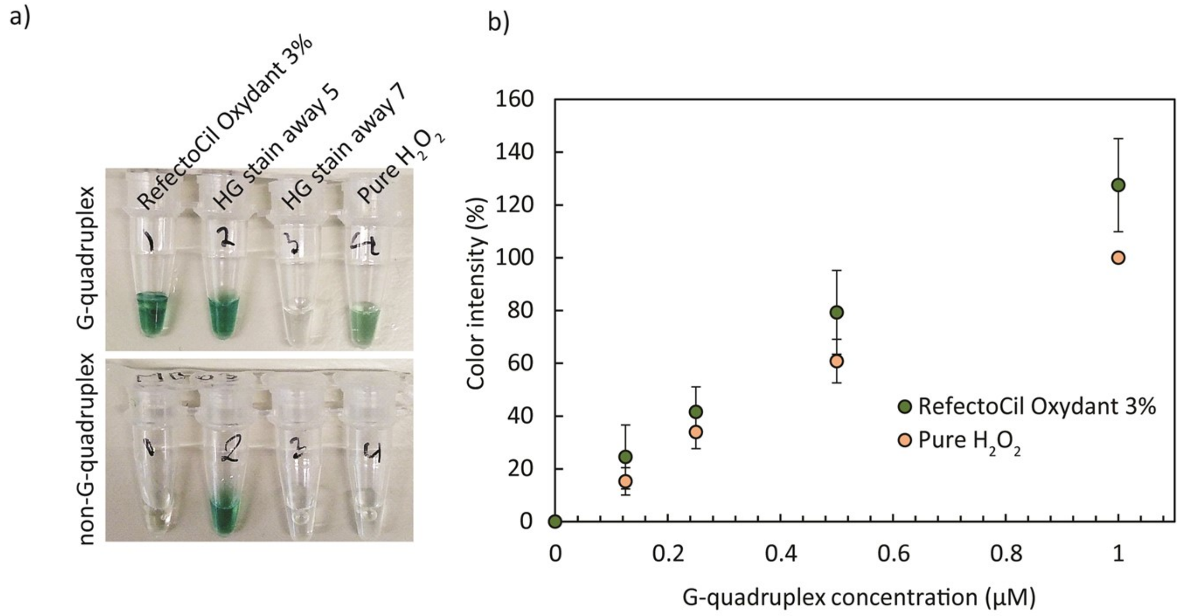

Supplementary Figure 8: Comparison of color production using pure H_2O_2 versus a commercial developer with H_2O_2

a) Comparing pure H_2O_2 to three alternative sources of H_2O_2, that are stable at room temperature, for color readout. Top panel contains G-quadruplex-forming oligonucleotide and the bottom panel contains non-G-quadruplex-forming oligonucleotide (see sequence in Supplementary Table 4.1). For the color reaction was performed in 1x phi29 buffer (33 mM Tris-acetate (pH 7.9 at 37 °C), 10 mM Mg-acetate, 66 mM K-acetate, 0.1% (v/v) Tween 20, 1 mM DTT) using a final concentration of 1 µM of the oligonucleotides, 4.7 µM hemin, 4.7 mM ABTS, 3.1 µL 40 mM pure H_2O_2 in a total of 27 µL (final 5.8 mM) or corresponding volume of other sources. b) The Y axis shows the normalized color intensity as a function of the concentration of the G-quadruplex-forming oligonucleotide on the X axis. Orange dots indicate the color intensity (%) with reference to using 8.67 µL 40 mM pure H_2O_2. Green dots indicate the color intensity (%) when using 8.67 µL of the RefectoCil Oxidant 3%, GW cosmetics. All data points have been normalized to give 100% color intensity using pure H_2O_2 with 1 µM G-quadruplexes. The results show that the H_2O_2-containing RefectoCil Oxidant yields a more intense color than 40 mM pure H_2O_2, in addition to being a more stable alternative source of H_2O_2 for PoC diagnostic tests for use in resource-limited settings.

```
>AJ270147.  Leishmania sp.                      CCCAAACTTTTCTGGTCCTCCGGG TAGGGGCGTTCTGCGAAGATGG   AAAAATGGGTG
>KM555295. L.major                              CC  AAACTTTTCTGGTCCTCCGGG TAGGGGCGTTCTGCGAA  ATCGG AAAAATGGGTG
>AF169137.  L.chagasi                           CC   AAACTTTTCTGGTCCTCCGGG TAGGGGCGTTCTGCGAAAATCG   AAAAATGGGTG
>AF184044.  L.infantum                          CC  AAACTTTTCTGGTCCTCCGGG TAGGGGCGTTCTGCGAAA TCGG AAAAATGGGTG
>AF168357.  L.donovani                          CCCAAACTTTTCTGGTCCTCCGGG TAGGGGCGTTCTGCAAAA TCGG AAAAATGGGTG
>AF103742.  L.donovani                          CCCAAACTTTTCTGGTCCTCCGGG TAGGGGCGTTCTGCAAAA TCGG AAAAATGGGTG
>AJ223724.  L.infantum kala-azar patient isolate CCCAAACTTTTCTGGTCCTTCGGG TAGGGGCGTTCTGCGAAAACC  GAAAAATGGGTG
>AJ010087.  L.donovani VL patient isolate       CCCAAACTTTTCTGGTCCTTCGGG TAGGGGCGTTCTGCGAAAA CC  GAAAAATGGGTG
>AJ270146.  Leishmania sp. blood isolate        CCCAAACTTTTCTGGTTCTTCGGG TAGGGGCGTTCTGCGAAAA  CC GAAAAATGGGTG
>Z35271.     L.infantum (AJS-IPTEC)             CCCAAACTTTTCTGGTCCTCCGGG TAGGGGCGTTCTGCGAAAA  CC  GAAAAATGGGTG
>M28567.     L.tarentolae                       CCCAAACTTTTTAGGTCCCTC AGG TAGGGGCGTTCTCCGAAAA  CC GAAAAAT GCATG
>KY698852.  L.amazonensis                       CCCAAACTTTTCTGCCCCGTGGGGGAGGGGCGTTCTGCGATTTTGG GAAAAATGGGTG
--------------------------------------------------------------------------------------------------------
>AJ270147.  Leishmania sp.                      CAGAAACCCCGTTCAAAAATCGGC CAAAAATGCCAAAAA TCGGCTCCGGGGCGGGAAA
>KM555295. L.major                              CAGAAATC CCGTTCAAAAATCGGC CAAAAATGCCAAAAA TCGGCTCCGGGGCGGGA
>AF169137.  L.chagasi                           CAGAAATC CCGTTCAAAAATCGGC CAAAAATGCCAAAAA TCGGCTCCGGGGCGGGAAA
>AF184044.  L.infantum                          CAGAAATC CCGTTCAAAAATCGGC CAAAAATGCCAAAAA TCGGCTCCGGGGCGGGAAA
>AF168357.  L.donovani                          CAGAAATC CCGTTCAAAAATCGGC CAAAAATGCCAAAAA TCGGCTCCGGGGCGGGAAA
>AF103742.  L.donovani                          CAGAAATC CCGTTCAAAAATCGGC CAAAAATGCCAAAAATCGGCTCCGGGGCGGAAAA
>AJ223724.  L.infantum kala-azar patient isolate CAGAAATC CCGTTCAAAAATCGGC CAAAAATGCCAAAAATCGGCTCCGGGGCGGGA
>AJ010087.  L.donovani VL patient isolate       CAGAAATCCCGTTCAAAAAA   TCC CAAAAATGCCAAAAATCGGCTCCGGGGCGGGAAA
>AJ270146.  Leishmania sp. blood isolate        CAGAAATCCCGTTCAAAAAATTG   CAAAAAATGCCAAAAATCGGCTCCGGGGCGGGAAA
>Z35271.     L.infantum (AJS-IPTEC)             CAGAAATCCCGTTCAAAAAATGTC  CAAAAA TGCCTAAAA TCAGCTCCG AGGCGGGAAA
>M28567.     L.tarentolae                       CAGAAACCCCGTTCAAAAATCGGC CAAAA
>KY698852.  L.amazonensis                       CAGAAACCCCGTTCA
```

Supplementary Figure 9: Target selection for the diagnosis of leishmaniases.

Overview (screenshot) of a multiple sequence alignment analysis that shows the unique identifying sequence code per *leishmania* species isolate (far left), and the resulting sequence (right) which is plotted across the top and bottom columns. Multiple alignment tool (T-coffee software, tcoffee.crg.cat) was used to identify a consensus region across the pan-*leishmania* genus that could serve as a potential target for detection by CRISPR-dCas9. Multiple iterations yielded putative targets within the kinetoplast minicircle DNA for recognition of *L. major, L. chagasi, L. infantum, L. donovani.* and *L. tarentolae.* Using BLAST, the identified targets were further checked for nonhomology with human or other pathogen's sequences. A conserved sequence of 115bp (full sequences denoted) was identified, that contains a 23-mer CRISPR-dCas9 target site (green, underlined) that has no homology with other genomes.

a)

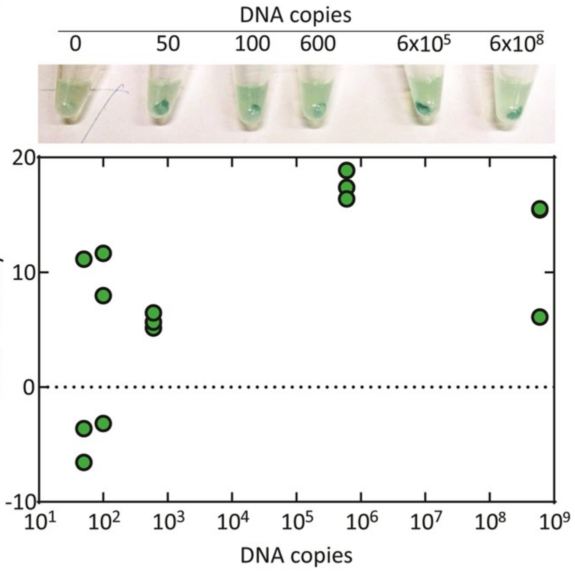

b)

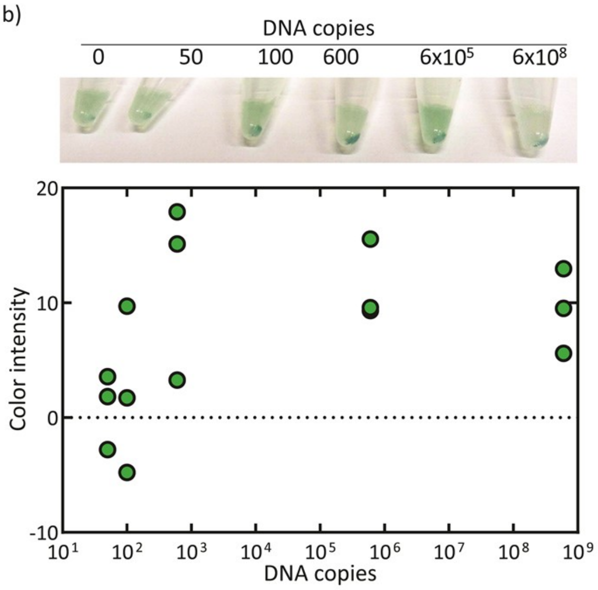

Supplementary figure 10. Data points from barcharts in Figure 5 represented as x-y plots.

a) Sensitivity of the assay using naked DNA. RPA reactions were spiked with DNA from 0 to 1 fmol of DNA molecules, followed by the remaining steps in the DNA-detection scheme. The top panel shows an image of the final reaction after the colorimetric readout. The chart below the image shows the color intensity of the precipitated beads from images of the tubes in three independent experiments, where background has been subtracted (n=3). b) Sensitivity of the full assay using simulated patient samples from DNA extraction with M-PER to the final color reaction in the tubes. The top panel shows an image of the test tubes after the colorimetric readout. The chart below shows quantification of the color intensity of the precipitated beads in the tubes in three independent experiments (n=3), where background has been subtracted. Color intensity of the beads was measured using ImageJ.

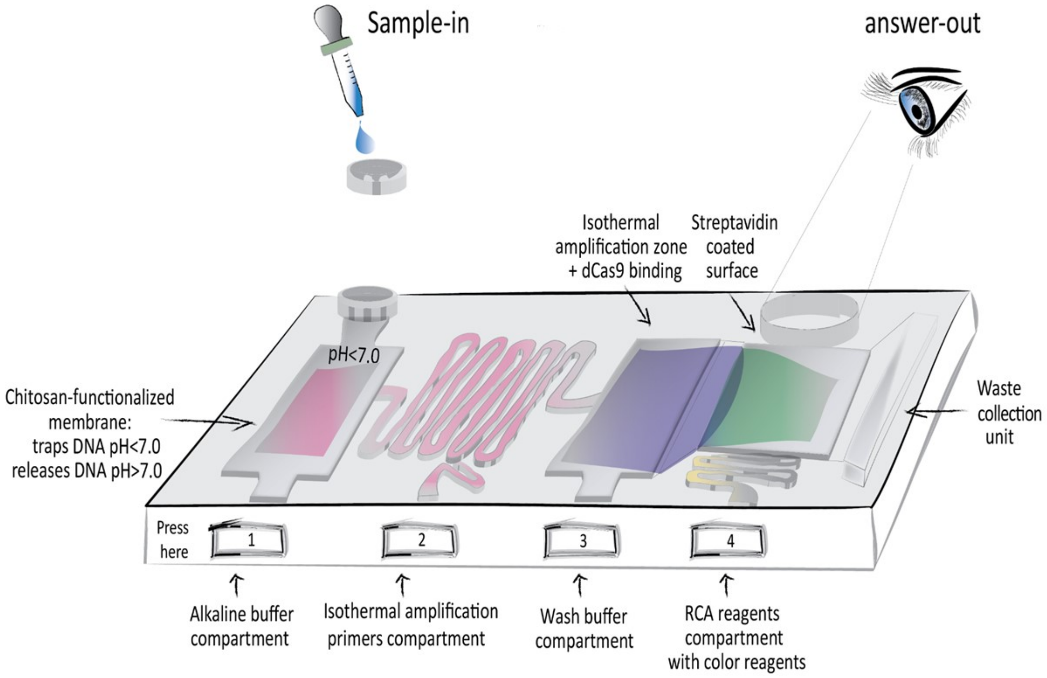

Supplementary Figure11: Possible microfluidic packaging of the DNA sensor as a rapid diagnostic test.

Sketch of how a simple DNA sensor with sample-in, answer-out capabilities could be packaged into a microfluidic device. Sn easy-to-perform test would come with an accurate binary result in the form of a color in the control and test zones that is visible to the naked eye. While a color in the control zone would ensure the functionality of the test, an appearance of the test color at the probing site would confirm a positive result, i.e., the presence of pathogen's DNA in the biological sample that was applied.

Supplementary Table 4.1: Oligonucleotide sequences used.

Synthetic *leishmania* target FWD primer	5’ CCCAAACTTTTCTGGTCCTCCG 3’
Synthetic *leishmania* target REV primer	5’ TTTCCCGCCCCGGAGC 3’
VL target FWD PCR primer	5’ GGGGCGTTCTGCGAAGA 3’
VL target REV PCR primer	5’ GCCCCGGAGCCGAT 3’
VL target FWD RPA primer	5’ CCCAAACTTTTCTGGTCCTCCGGGTAGGGGC
VL target REV RPA primer	5’ TTTCCCGCCCCGGAGCCGATTTTTGGCATT
Biotinylated VL target FWD RPA primer	5’ Biotin/TTTTTTGAATTCCCCAAACTTTTCTGGTCCTCCGGGTAGGGGC 3’
sgRNA FWD primer	5’ TAATACGACTCACTATAGGCAGAAACCCCGTTCAAAAATGTTTTAGAGCTAGAAATAGCAAGTTAAAATAAGG 3’
sgRNA REV primer	5’ AAAAAAAGCACCGACTCGGTGCCAC 3’
RCA01	5’ TTTTTTTTTTTACATGCTCGAGATCAGTTTTTTATGCGCCTGTTGCC 3’
RCA02	5’ CTACTACCTCACCTCACCCAACCCGCCCTACCCAAAACCCAACCCGCCCTACCCAAAACCCAACCCGCCCTACCCAAAAGGCAACAGGCGCATAAAACAACTATACAAC 3’
RCA03	5’ GAGGTAGTAGGTTGTATAGT 3’
RCA04	5’ CTACTACCTCACCTCACCCAACCCGCCCTACCCAAAACCCAACCCGCCCTACCCAAAACCCAACCCGCCCTACCCAAAAGGCAACAGGCGCATAAAACACCTCAGCACTATACAAC 3’
G-quadruplex forming oligonucleotide	5’ GTTGTATAGTTGTTTTATGCGCCTGTTGCCTTTTGGGTAGGGCGGGTTGGGTTTTGGGTAGGGCGGGTTGGGTTTTGGGTAGGGCGGGTTGGGTGAGGTGAGGTAGTAG 3’
non-G-quadruplex forming oligonucleotide	5’ CCCAACCCGCCCTACCCAAAACCCAACCCGCCCTACCCAAAACCCAACCCGCCCTACCCAAAA 3’
